# Dengue and Zika virus capsid proteins bind to membranes and self-assemble into liquid droplets with nucleic acids

**DOI:** 10.1016/j.jbc.2021.101059

**Published:** 2021-08-08

**Authors:** Ernesto E. Ambroggio, Guadalupe S. Costa Navarro, Luis Benito Pérez Socas, Luis A. Bagatolli, Andrea V. Gamarnik

**Affiliations:** 1 Departamento de Química Biológica Ranwel Caputto, Facultad de Ciencias Químicas, CIQUIBIC, CONICET, Universidad Nacional de Córdoba, Córdoba, Argentina; 2 Fundación Instituto Leloir-CONICET, Buenos Aires, Argentina; 3 Instituto de Investigación Médica Mercedes y Martín Ferreyra, INIMEC (CONICET), Universidad Nacional de Córdoba, Córdoba, Argentina; 4 Departamento de Química Biológica Ranwel Caputto, Facultad de Ciencias Químicas, Universidad Nacional de Córdoba, Córdoba, Argentina

**Keywords:** Zika and dengue, flavivirus, capsid–membrane interaction, liquid–liquid phase separation, capsid–RNA interaction, ACDAN, 6-acetyl-2-dimethylaminonaphthalene, DENV, dengue virus, E, envelope, ER, endoplasmic reticulum, GUV, giant unilamellar vesicle, HKM, Hepes buffer with 120 mM potassium acetate and 1 mM MgCl_2_, L_d_, liquid disordered, LR, ligand–receptor complex, LUV, large unilamellar vesicle, NBD, nitrobenzoxadiazole, NC, nucleocapsid, PB, phosphate buffer, PE, phosphatidylethanolamine, prM, pre-Membrane, TRITC, *N*-(teramethylrhodamine-6-thiocarbamoyl), W, tryptophan, ZIKV, Zika virus

## Abstract

Dengue virus (DENV) and Zika virus (ZIKV) capsid proteins efficiently recruit and surround the viral RNA at the endoplasmic reticulum (ER) membrane to yield nascent viral particles. However, little is known either about the molecular mechanisms by which multiple copies of capsid proteins assemble into nucleocapsids (NCs) or how the NC is recruited and wrapped by the ER membrane during particle morphogenesis. Here, we measured relevant interactions concerning this viral process using purified DENV and ZIKV capsid proteins, membranes mimicking the ER lipid composition, and nucleic acids in *in vitro* conditions to understand the biophysical properties of the RNA genome encapsidation process. We found that both ZIKV and DENV capsid proteins bound to liposomes at liquid-disordered phase regions, docked exogenous membranes, and RNA molecules. Liquid–liquid phase separation is prone to occur when positively charged proteins interact with nucleic acids, which is indeed the case for the studied capsids. We characterized these liquid condensates by measuring nucleic acid partition constants and the extent of water dipolar relaxation, observing a cooperative process for the formation of the new phase that involves a distinct water organization. Our data support a new model in which capsid–RNA complexes directly bind the ER membrane, seeding the process of RNA recruitment for viral particle assembly. These results contribute to our understanding of the viral NC formation as a stable liquid–liquid phase transition, which could be relevant for dengue and Zika gemmation, opening new avenues for antiviral intervention.

Proteins in solution can phase separate into liquid compartments by intermolecular interactions with nucleic acids. Such transitions occur with a first seed formed either in bulk or at the interface of substrates/soluble components, leading to a new liquid entity that displays different physical properties compared with those of its surroundings. The nucleolus is the paradigm of these liquid compartments, nowadays known as membrane-less organelles, but already described in the 1830s (see Refs. (1, 2) and references therein). It is accepted that one of the *in vivo*–*in vitro* interaction that triggers liquid–liquid phase separation is the association of RNA with proteins. One mechanism is by enabling multimerization of the RNA-binding protein (3). Furthermore, there are several reports indicating that proteins can also self-phase separate into liquid phases at the lipid bilayer interface (4). The capsid proteins of dengue virus (DENV) and Zika virus (ZIKV) interact with viral RNA, forming a nucleocapsid (NC), in the process of viral genome packaging. This process is called encapsidation, and up to date, a clear notion concerning the molecular mechanisms behind this phenomenon is not fully understood (5, 6).

DENV and ZIKV are relevant mosquito-borne human pathogens of the *Flavivirus* genus. Upon infection, the positive-stranded RNA genome is directly used as a messenger for translating a large polyprotein, which is proteolytically processed at the endoplasmic reticulum (ER) membrane. The viral capsid is the first protein encoded in the open reading frame and remains inserted into the ER membrane by a transmembrane anchor peptide that links capsid to the pre-Membrane (prM) protein. Two successive cleavages exerted by cellular and viral proteases are necessary for capsid release. The viral protease NS2B/NS3 is responsible for cleaving the anchor peptide, permitting the release of the mature capsid protein at the cytoplasmic side (5, 6). Capsid proteins recruit the newly synthesized viral genome to form the NC complex, which subsequently buds into the ER lumen gaining the lipid bilayer together with the viral proteins envelope (E) and prM (5). How NC is recruited to the ER membrane is under extensive scrutiny. So far, high-resolution microscopy techniques such as cryo-EM do not provide compelling evidence that the capsid or NC is physically associated to the inner leaflet of the viral lipid membrane (7, 8, 9). To attempt to answer these questions, we use fluorescence methods to measure the interaction of both DENV and ZIKV capsids with ER-mimicking lipid membranes in the absence or in the presence of nucleic acids. We describe how ZIKV and DENV capsid proteins interact with liposomes and RNA molecules. We measured the protein–membrane binding and the ability of DENV and ZIKV capsids to recruit ssDNA or RNAs and liposomes onto the interface of giant unilamellar vesicles (GUVs). The capsid–RNA–membrane association was found at regions corresponding to liquid disordered (L_d_) phase. Also, the viral protein–RNA interaction generated phase-separated liquid droplets, with clear-cut changes on water organization within the droplets respect to their surroundings. We further characterized this process by computing an apparent dissociation constant for the nucleic acids–protein interaction, together with the determination of the cooperativity of the process.

## Results

### DENV and ZIKV capsid proteins bind and dock liposomes

**Figure 1:**
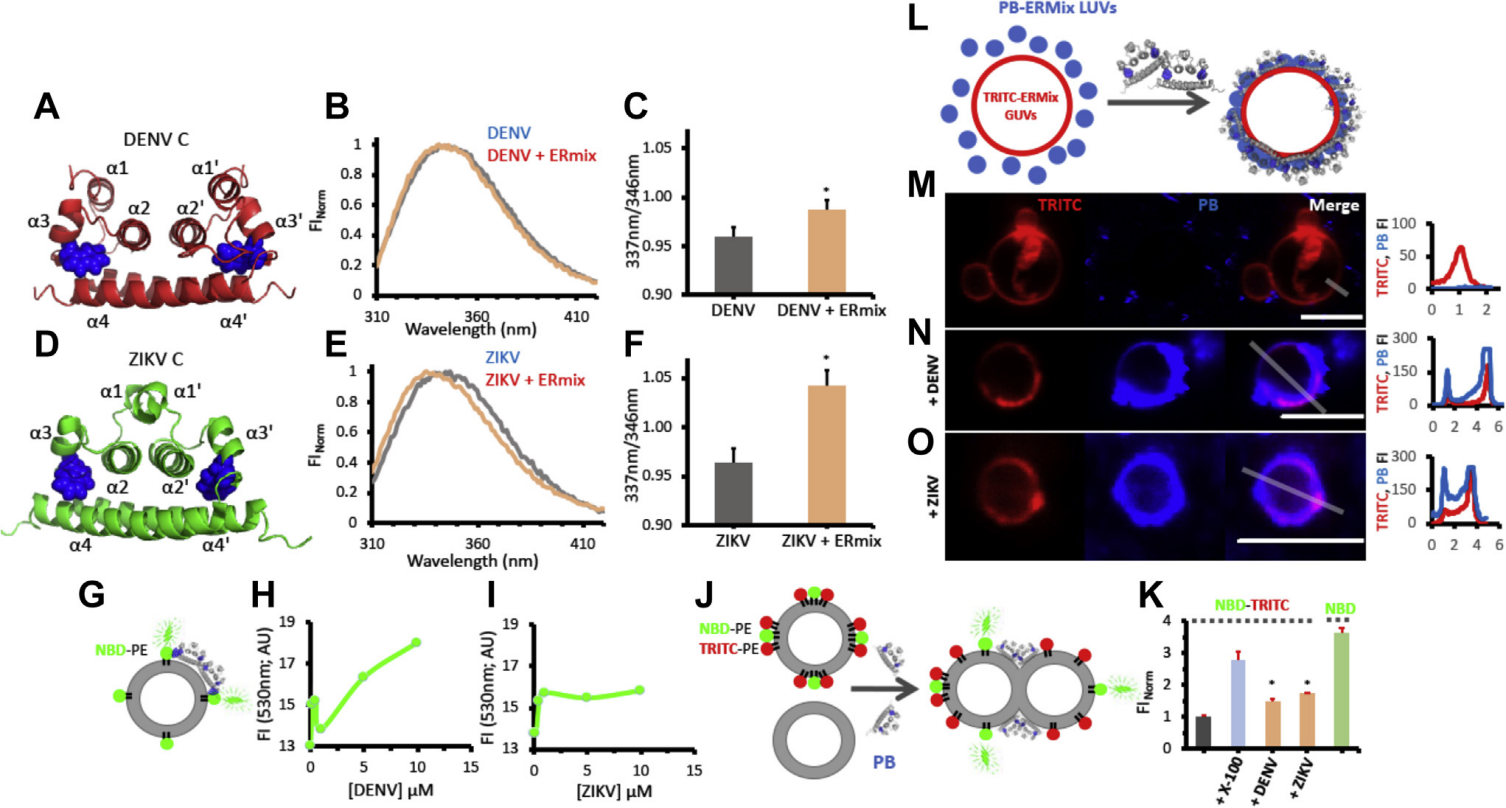
**DENV and ZIKV capsid interaction with ERmix liposomes.** The dimeric structures of DENV (*A*) and ZIKV (*D*) capsids, where the W residue is represented in *blue spheres*. W fluorescence emission spectra and quantification of the 337/346 nm wavelength ratio for DENV (*B* and *C*) and ZIKV (*E* and *F*), respectively, in the absence (*gray*) and presence (*light brown*) of ERmix LUVs. *Cartoon* representing W–NBD FRET (*G*) when capsid proteins interact with NBD–PE labeled ERmix LUVs. The increase in NBD fluorescence intensity (FI) because of W proximity (λ_ex_ = 280 nm; λ_em_ = 530 nm) is measured in function of DENV (*H*) or ZIKV (*I*) addition. The lipid mixing assay represented in *J*, where NBD fluorescence (donor dye) increases when lipid content from nonlabeled LUVs fuses with NBD–PE/TRITC–PE dyed LUVs. *K*, the normalized FI of NBD of the mixture NBD–PE/TRITC–PE stained ERmix LUVs + ERmix LUVs alone for the different conditions is shown. A GUVs–LUVs docking assay triggered by protein addition is schematized in *L*, where *blue-fluorescent* LUVs are recruited to the membrane of *red-fluorescent* GUVs after addition of the capsid proteins. Confocal fluorescence images of the ERmix GUVs labeled with TRITC–PE (*red channel*; TRITC) in the presence of ERmix LUVs doped with PB–PE (*blue channel*, PB) in the absence (*M*) or in the presence of DENV (*N*) or ZIKV (*O*) capsids are shown. From the color merge image (Merge), the TRITC (*red line*) and PB (*blue line*) FI profile was measured along the drawn *gray line* and plotted at the *right* of each micrography. Note that the PB fluorescence signal profiles plotted in *N* and *O* reach their maximum values since images are acquired with exactly same settings as the control (*M*). Results are from at least two independent experiments (quantitative plots showing average and standard deviations where ∗ indicate a statistically significant difference with respect to the control situation using the unpaired Student's *t* test), and images show representative observations. The scale bar in confocal images is 5 μm. Data shown are representative of 2 to 4 independent replicates. DENV, dengue virus; ER, endoplasmic reticulum; GUV, giant unilamellar vesicle; LUV, large unilamellar vesicle; NBD, nitrobenzoxadiazole; PB, phosphate buffer; PE, phosphatidylethanolamine; TRITC, *N*-(teramethylrhodamine-6-thiocarbamoyl); W, tryptophan; ZIKV, Zika virus.

To investigate whether DENV and ZIKV capsid proteins interact with membranes, we used different fluorescence methods. We first monitored the characteristics of the fluorescence emission spectra of a single tryptophan (W) contained in the proteins (highlighted in Fig. 1, *A* and *D*) in the absence and presence of ERmix large unilamellar vesicles (LUVs). W fluorescence emission properties are well known to depend on the polarity of the environment (10). The W emission spectra of both DENV and ZIKV capsids show a shift toward shorter wavelengths (Fig. 1, *B* and *E*) in the presence of LUVs, which is quantified by the ratio of the fluorescence intensities at 337 and 346 nm (Fig. 1, *C* and *F*). This indicates that upon membrane interaction, the W senses a less polar milieu. The polarity change can be associated to a protein structural rearrangement, to a new environment because of the proximity of the amino acid to the membrane, or both. To answer this question, we took advantage of the well-known energy transfer effect between W and the nitrobenzoxadiazole (NBD) dye, represented in Figure 1*G* (11). The fluorescence intensity of NBD rises upon titration of NBD-dyed ERmix LUVs (where NBD is at the interface of the membrane) with increasing amounts of DENV and ZIKV capsid proteins (Fig. 1, *H* and *I*), respectively. These data indicate that proteins bind to membranes, and their Ws are sufficiently close to the lipid bilayer interface (2.2 nm (12)) to transfer excited state energy to NBD. In addition, the lipid–protein interaction was corroborated either by measuring electrophoretic mobility shift of ERmix LUVs (13) or by performing lipid sedimentation assays (Supporting information).

During the aforementioned experiments, a change in the turbidity of the sample was observed, suggesting that protein interaction with the liposomes may induce a proteoliposome aggregation. To further explore whether this process involved lipid mixing between different liposomes (*i.e.*, throughout fusion or hemifusion of LUVs), we performed a FRET assay using the NBD–phosphatidylethanolamine (PE)/*N*-(teramethylrhodamine-6-thiocarbamoyl) (TRITC)–PE couple, as schematized in Figure 1*J* (see Experimental procedures for more details). Upon addition of either DENV or ZIKV capsid proteins to the labeled/nonlabeled liposome mixture, an increase of NBD fluorescence signal about 1.5 times higher than the control (normalized to 1) was observed, indicating lipid exchange among LUVs. Interestingly, this change represents on average half of the full intensity registered with respect to controls (Fig. 1*K*). These data support the idea that hemifusion is occurring upon protein–membrane interaction. In order to achieve spatially resolved information about this interaction, we used confocal fluorescence microscopy to visualize directly the membrane docking between ERmix GUVs and ERmix LUVs. For this, TRITC-labeled GUVs were incubated with pacific blue–stained LUVs in the absence or the presence of viral capsid proteins. In the absence of proteins, there was no LUVs signal at the membrane of GUVs (Fig. 1*M*). However, upon addition of DENV (Fig. 1*N*) or ZIKV (Fig. 1*O*) capsids, there is a clear recruitment and enrichment of LUVs at the GUVs membrane interface, where a strong spatial colocalization of the two dyes was observed.

### DENV and ZIKV recruit liposomes and nucleic acids at the GUV membrane interface

**Figure 2:**
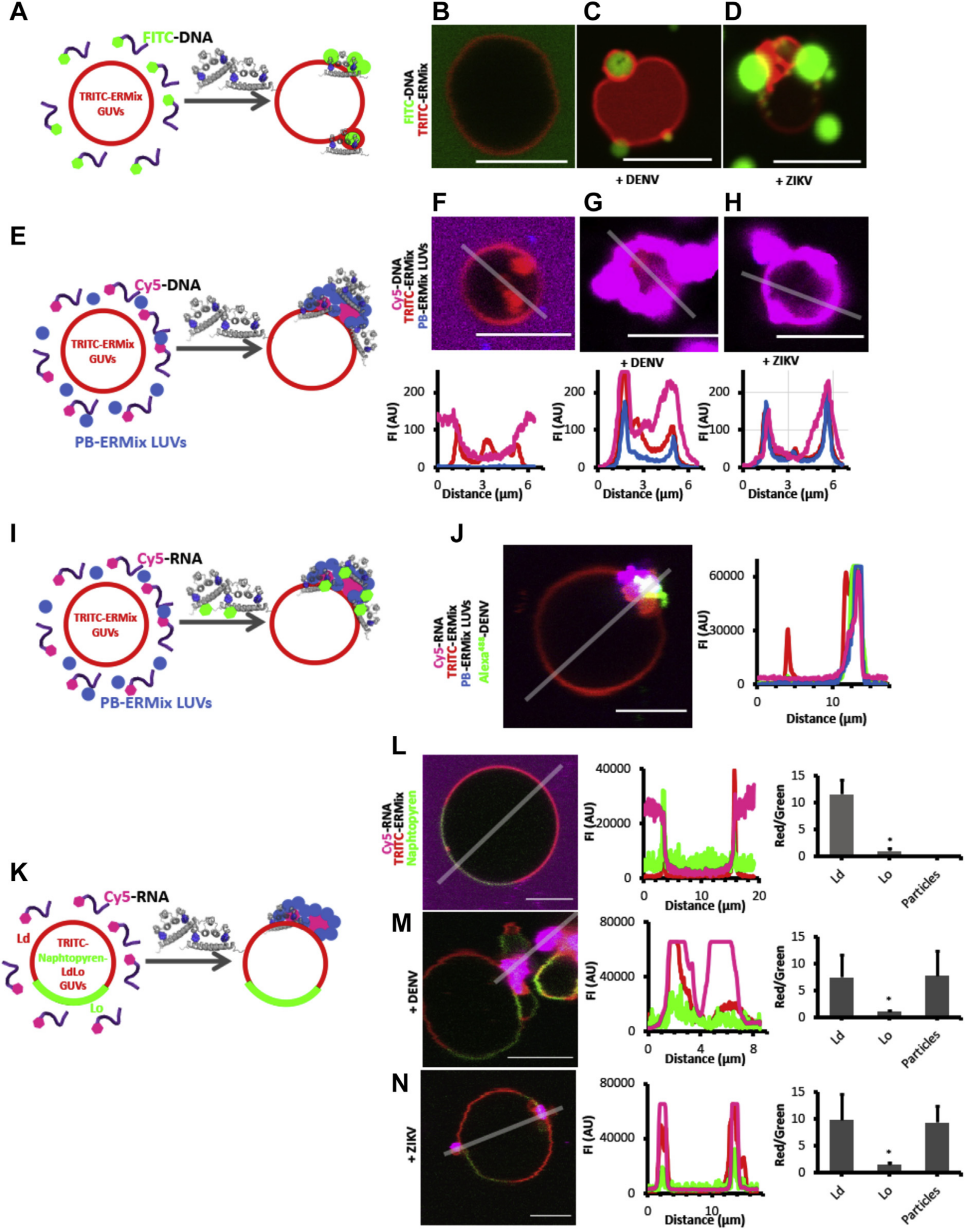
**DENV and ZIKV capsids interact with ERmix GUVs and recruit to the lipid membrane LUVs and oligonucleotides.** Schematic representation of FITC-labeled ssDNA recruitment at the membrane of ERmix GUVs doped with TRITC–PE after capsid protein addition (*A*). Representative color-merged confocal fluorescence images of TRITC–PE ERmix GUVs (*red channel*) incubated with FITC–DNA (*green channel*) in the absence (*B*) or in the presence of DENV (*C*) or ZIKV (*D*) capsids denote the particular recruitment of the labeled oligonucleotide by the capsids. Protein-triggered liposome and oligonucleotide recruitment to the GUV membranes is *cartooned* in *E*. Representative color-merged confocal fluorescence images of TRITC–PE ERmix GUVs (*red channel*) incubated with Cy5-DNA (*magenta channel*) and with PB-labeled ERmix LUVs, in the absence (*F*, *upper image*) or in the presence of DENV (*G*, *upper image*) or ZIKV (*H*, *upper image*) capsids. The *lower panels* of *F*–*H* show the fluorescence intensity profile of the *red*, *magenta*, and *blue channels* along the *gray line*. Alexa^488^-DENV also causes recruitment of oligonucleotide and LUVs to the membrane of GUVs (*I*). Representative color-merged confocal fluorescence images of TRITC–PE ERmix GUVs (*J*, *red channel*) incubated with Cy5-DNA (*J*, *magenta channel*) and with PB-labeled ERmix LUVs (*J*, *blue channel*) after addition of Alexa^488^-DENV (*J*, *green channel*). The *right panel* in *J* shows the FI profile of the *red*, *magenta*, *blue*, and *green* channels along the *gray line* (control condition and additional data of this condition are presented in the Supporting information section). DENV and ZIKV capsids recruit CY5-labeled RNA at L_d_ regions of the membrane of GUVs with L_o_/L_d_ lipid phases (*K*). Representative color-merged confocal fluorescence images of TRITC–PE (*J*, *red channel*, Ld marker) and naphthopyrene (*J*, *green channel*, Lo marker) labeled L_o_/L_d_ GUVs incubated with Cy5-DNA (*J*, *magenta channel*) before (*L*) and after addition of DENV (*M*) or ZIKV (*N*) capsid proteins. The *middle panels* in *L*–*N* show the FI profile of the *red*, *magenta*, and *green channels* along the depicted *gray line*. Quantitative bar plots at the right panels of *L*–*N* show the *red/green* FI ratio for L_d_ and L_o_ regions and that at the membrane region where Cy5-RNA particles are observed. Note that some fluorescence signal profiles plotted reach their maximum values because images are acquired with exactly same settings as the control situation. Results are from at least two independent experiments (quantitative plots showing average and standard deviations where ∗ indicates a statistically significant difference with respect to the plotted values of the unpaired Student's *t* test), and images show representative observations. The scale bar in confocal images scales to 5 μm. Data shown are representative of 2 to 4 independent replicates. DENV, dengue virus; ER, endoplasmic reticulum; GUV, giant unilamellar vesicle; L_d_, liquid disordered; LUV, large unilamellar vesicle; PB, phosphate buffer; PE, phosphatidylethanolamine; TRITC, *N*-(teramethylrhodamine-6-thiocarbamoyl); ZIKV, Zika virus.

DENV and ZIKV capsids form the NC by binding to the viral RNA genome; however, it is still unknown how the NC is recruited into the nascent viral particle at the ER membrane. The NC must get in close contact with ER membranes for particle morphogenesis, but it is uncertain whether capsid–lipid membrane interaction is a driving force in this crucial step. To further analyze the role of the interaction with lipid membranes of the DENV and ZIKV capsid proteins, we carried out an experiment where ERmix GUVs were incubated with a fluorescently labeled ssDNA or ssRNA in the presence or the absence of such proteins (Fig. 2). In their absence, the green labeled nucleic acid was homogeneously distributed (Fig. 2*B*); however, in their presence, the nucleic acid was massively recruited at the membrane interface of GUVs (Fig. 2, *C* and *D*). As observed in the images, the recruitment was not uniform on the surface of the GUVs, and DNA-stained particles sizing 1 to 2 μm diameter were either bound to or surrounded by membranes (Fig. 2, *C* and *D*). In addition, when labeled LUVs and ssDNA were present, the nucleic acid and liposomes were both targeted to the interface of GUVs mediated by the capsid proteins (Fig. 2, *F*, *G* and *H*: control before protein addition).

Furthermore, to better characterize the system, the capsid proteins were labeled. Adding Alexa^488^-cysDENV to the cosuspension of ERmix GUVs, Cy5 fluorescently labeled RNA and small phosphate buffer (PB)–stained LUVs, the same phenomena were observed at the interface of GUVs, confirming that the viral proteins trigger and are contained in these supramolecular aggregates (Fig. 2, *I* and *J*). These observations are particularly relevant because they indicate that DENV and ZIKV capsid proteins can spontaneously recruit nucleic acids and membranes onto the interface of a targeted membrane.

Finally, when GUVs display L_o_/L_d_ domains, the RNA/DNA-containing particles are localized at regions corresponding to L_d_ phases (*red channel*, Fig. 2, *L*–*N*), suggesting a site-specific membrane association when capsid proteins recruit RNA. The experiments highlight that the process involves a large amount of material clustered as a puncta or a droplet shape and is always enhanced when nucleic acids are present. These aggregates resemble liquid condensates as reported for several similar situations when positively charged (proteins) and negatively charged (nucleic acids) polymers interact (14, 15, 16).

### DENV and ZIKV conform reversible liquid condensed droplets when interacted with RNA

**Figure 3:**
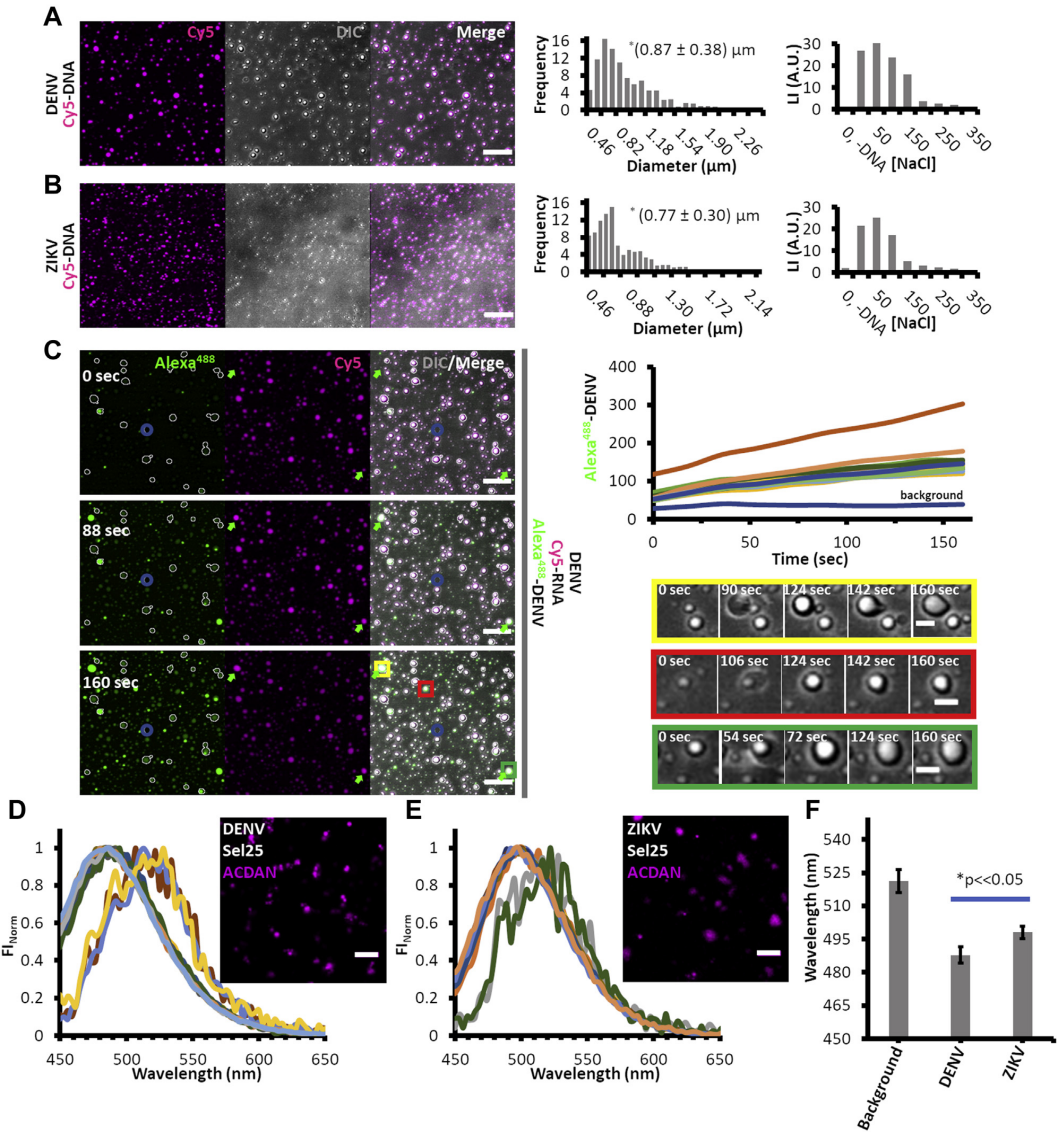
**DENV and ZIKV capsid phase-separate into liquid droplets when bind oligonucleotides.** Representative confocal fluorescence images of Cy5-DNA (*magenta*) incubated with DENV (*A*, *left panel*) or ZIKV (*B*, *left panel*) from where the fluorescence oligonucleotide colocalizes with spherical particles observed by DIC (*gray channel*). From the images, the histogram of the particle size distribution frequency can be measured as represented in the *middle plots* of *A* and *B*. At the *right panel* of *A* and *B*, nephelometry measurements (see Experimental procedures section) are reported showing scattered-light intensity *versus* NaCl concentration when DENV (*A*) or ZIKV (*B*) capsids phase-separate when interact with DNA depicting the reversibility of the phase-separation process. Incubation of DENV and Cy5-RNA (*C*, *magenta*) develops Cy5-positive droplets that can recruit exogenous injected Alexa^488^-DENV (*C*, *green*) in a time-dependent manner as shown in the *upper right panel* where the fluorescence intensity of Alexa^488^-DENV increases at the ROIs where Cy5 positive particles are present (*C*, *upper right plot*). From these representative experiments, several situations can be identified (*yellow*, *red*, and *green rectangles* at the *bottom right panel* of *C* showing DIC images of the respective *colored squares* in the color-merge panel at a time point of 160 s) where new particles are generated and fuse with neighboring particles (*yellow*) or particles grow from a pre-existing seed (*red* and *green*). Water structure in the bulk or at the region of the droplets is measured with spectral confocal fluorescence microscopy (images in *inset* of *D* and *E*; see Experimental procedures section) taking advantage of the sensibility of the ACDAN dye to water dipolar relaxation. In this sense, a spectral *blue shift* is observed for the emission spectrum of ACDAN colocalizing with DNA-stabilized DENV (*D*) or ZIKV (*E*) particles respect to the sample solution. Quantitative analysis of the spectral imaging (*F*) evidences a differential maximum emission wavelength of each condition (background: no particles and DENV or ZIKV droplets). Results are from at least two independent experiments (quantitative plots showing average and standard deviations), and images show representative observations. The scale bars in confocal images scale to 5 μm (*A* and *C*, *left panel*; *D* and *E*) and 1 μm (*C*, *bottom right panel*). ∗ in *F* represents a significant statistical difference between the compared values evaluated with a *t* test of two-sample assuming equal variances (α = 0.05). Data shown are representative of 2 to 4 independent replicates. ACDAN, 6-acetyl-2-dimethylaminonaphthalene; DENV, dengue virus; DIC, differential interference contrast; ROI, region of interest; ZIKV, Zika virus.

When nucleic acids interact with the viral capsid proteins, we regularly observed an increase of turbidity and spherical aggregates attached to the glass (like those noticed to be associated to GUVs). Particles showing either DNA or RNA can be observed thorough the fluorescence microscope showing a broad size distribution (Fig. 3, *A* and *B*, *left and middle panels*). DENV particles interacting with Cy5-DNA present an average diameter of 0.87 μm, whereas, in the same conditions, ZIKV particles present an average diameter of 0.77 μm. Comparing their size and shape, these type of condensates correlate to self-assembled nucleic acid–driven liquid droplets (3). Therefore, we further explored the phase-separation process and analyzed the reversibility and physicochemical properties of the new phase. For example, the aggregate assembly can be reverted by increasing the ionic strength of the environment. This is shown in the quantitative bar plots at the *right panel* in Figure 3, *A* and *B*, where at low NaCl concentration (<150 mM), the turbidity of the samples containing either DENV or ZIKV capsid proteins and nonlabeled DNA (25 mer) is high, indicating the presence of particles that can scatter light, whereas this effect is diminished above this concentration. Control experiments (not shown) with heparin, a well-known liquid droplet stabilizer (17), are in line with the salt-dependent reversibility of the phase separation induction by polyanionic molecules (*i.e.*, RNA or DNA). Once formed, these self-assembled drops can recruit an exogenously added protein. This is the case when Alexa^488^-cysDENV is injected into a chamber containing DENV/Cy5-RNA–stabilized particles (Fig. 3*C*). Not only all particles became labeled by the fluorescent version of the capsid (Fig. 3*C*, *left* and *upper right panel*) but also some newly formed droplets stick together or appear from an already existing seed (Fig. 3*C*, *lower right panel*). This kind of dynamism is a key characteristic for such entities, proposed to be a reservoir of several biomacromolecules (3).

We also explored whether the extent of water dipolar relaxation is different in this new phase compared with that observed in bulk water. For this purpose, we used the polarity-sensitive dye 6-acetyl-2-dimethylaminonaphthalene (ACDAN), whose fluorescent properties largely depend on the dipolar relaxation of water in their vicinity (18, 19), a parameter connected to the rotational dynamic of water. Figure 3, *D* and *E* shows the change of the fluorescence emission spectrum of ACDAN at the interior of DNA-stabilized DENV and ZIKV particles, respectively, with respect to the solution, measured by spectral fluorescence microscopy. There is a noticeable shift of the ACDAN maximum emission intensity (Fig. 3*F*) from 521 ± 5 nm (determined from regions of interest where the dye is outside the particles) to 488 ± 3 nm (DENV) and 498 ± 2 (ZIKV) (determined from regions of interest where the dye is at the particles), indicating an important change of the extent of water relaxation in the droplets. Of notice is the statistically significant difference of the values found for ZIKV particles compared with those from DENV particles, suggesting a differential capacity to affect water by each capsid conforming the droplets.

### Thermodynamics of the induced liquid–liquid phase transition when nucleic acid interacts with DENV and ZIKV capsid proteins

**Figure 4:**
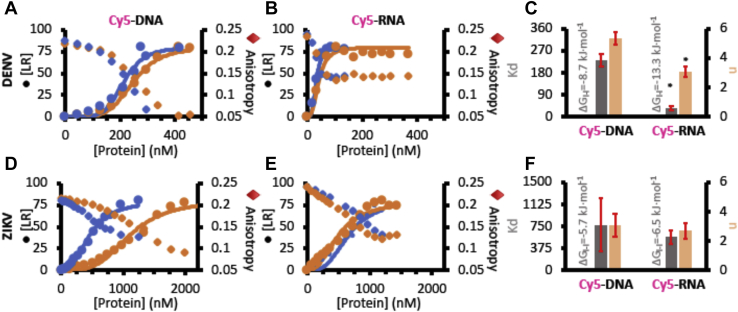
**Thermodynamics of DENV and ZIKV interaction with oligonucleotides assessed by fluorescence anisotropy.** Concentration of oligonucleotide–protein complex ([LR], *circles*) and anisotropy (*diamond*) changes of Cy5-DNA (*A* and *D*) and Cy5-RNA (*B* and *E*) in function of DENV (*A* and *B*) or ZIKV (*D* and *E*) concentration. *Blue* and *orange* data represent different individual experiments, and *solid lines* in *A*, *B*, *D*, and *E* are the experimental data fitting to the Hill equation (see Experimental procedures section). From fitting the dissociation constant, *K*_*d*_ (*C* and *F*; *gray bars*) and the Hill coefficient, n (*C* and *F*; *light brown bars*) are obtained for the interaction of Cy5-DNA (*C* and *F*, *left bar set*) and Cy5-RNA (*C* and *F*, *right bar set*) with DENV (*C*) or ZIKV (*F*) capsid proteins. Shown results are two independent experiments (quantitative plots showing average and standard deviations). ∗ in *C* represents a significant statistical difference between the compared values evaluated with a *t* test of two-sample assuming equal variances (α = 0.05). Data shown are representative of 2 to 4 independent replicates. DENV, dengue virus; LR, ligand–receptor; ZIKV, Zika virus.

To shed light into the thermodynamics of the liquid–liquid phase transition for the nucleic acid–capsid interaction, we took advantage of fluorescence anisotropy measurements of Cy5-labeled oligonucleotides. As previously reported, an increase in the turbidity of the sample can originate a decrease in anisotropy (20), an effect that can explain our results (homotransfer effects in the samples are discarded, see Experimental procedures section). Figure 4 summarizes the Cy5-DNA anisotropy change upon titration with DENV or ZIKV capsids. From these data, the fraction of adsorbed nucleic acid into the new phase is estimated. This allows not only to calculate relevant thermodynamic parameters of the process (association constant and free energy) but also to understand its cooperativity by using the Hill equation (see Experimental procedures section). Quantification from anisotropy data related to the concentration of DNA/RNA–protein complex formation (ligand–receptor [LR] complex) upon protein addition (Fig. 4, *A*, *B*, *D*, and *E*) confirms that the nuclei acid–capsid association and the aggregation process is a cooperative event, since Hill coefficients (n) are higher than 1 (Fig. 4, *C* and *F*). We obtained apparent dissociation constants (*K*_*d*_) in the order of 10^2^ nM, in agreement with those found, for example, for aptamers of proteins obtained by the SELEX methodology (21). From these data, the free energy of the transition process (ΔG_l−l_), both for DNA and RNA interacting with DENV and ZIKV capsids, can be estimated (Fig. 4, *C* and *F*). Our data show an exergonic process taking place in both cases.

## Discussion

Maturation of dengue and Zika viral particles at the membrane of the ER is a coordinated event where viral RNA is tightly packaged by capsid proteins and wrapped by the ER membrane before exiting the cell. So far, the NC approximation and ER association is proposed to be by protein–protein interaction with prM and E proteins at this organelle (5, 22). Recent data using ZIKV virus have shown that the transmembrane anchor peptide following the capsid protein may be partially retained on some molecules promoting oligomerization at the ER membrane, interaction with transmembrane regions of prM and E proteins, and consequent stabilization of particle assembly (23). Undoubtedly, the viral particle interior conforms a dense and condensed liquid phase as the result of the aforementioned interactions. Comprehension on how this phase is stabilized and how it is specifically localized at the correct organelle is essential to characterize virus development and approach crucial steps in where the viral morphogenesis can be blocked. In this sense, we studied how DENV and ZIKV capsids can interact with membrane model systems that mimic the ER lipid composition and the effect of the presence of DNA–RNA molecules and small liposomes along this association.

We find that both viral capsids can bind liposomes to promote membrane mixing, probably as a result of membrane hemifusion events. Even more, this membrane recruitment is observed together with DNA–RNA binding onto the interface of giant liposomes. The associated capsids on the lipid bilayer dock both small LUVs and exogenously added nucleic acids, generating a massive local membrane concentration at the surface of giant liposomes. Of note is that the protein–nucleic acid–lipid condensations were localized at the L_d_ domains of GUVs, showing a preferred lipid physical state for a potential viral particle formation. In addition, we describe how DENV and ZIKV capsids phase separate when bind to DNA or RNA. In this regard, we observed that the new phases display a droplet shape that can recruit extra-added material, such as the fluorescent version of DENV capsid or labeled nucleic acids. By analyzing the extent of water dipolar relaxation inside these droplets with ACDAN, we observed a slight spectral difference between DENV and ZIKV droplets. Specifically, the overall extent of water relaxation sensed by ACDAN in the case of DENV containing droplets is reduced with respect to ZIKV case. These differences may be due to structural capsid dissimilarities (7, 8, 9) and, in consequence, compositional features of these two proteins, which differentially impact in their ability to structure water (24). In fact, ZIKV capsids seem to be more flexible than those of the DENV protein, and this may impact on the water dipolar relaxation inside the capsid droplets. Furthermore, the thermodynamics of the liquid–liquid transitions reflects a spontaneous and a very cooperative process where a tight interaction of the oligonucleotides (in the order of 10–10^2^ nM) is observed.

**Figure 5:**
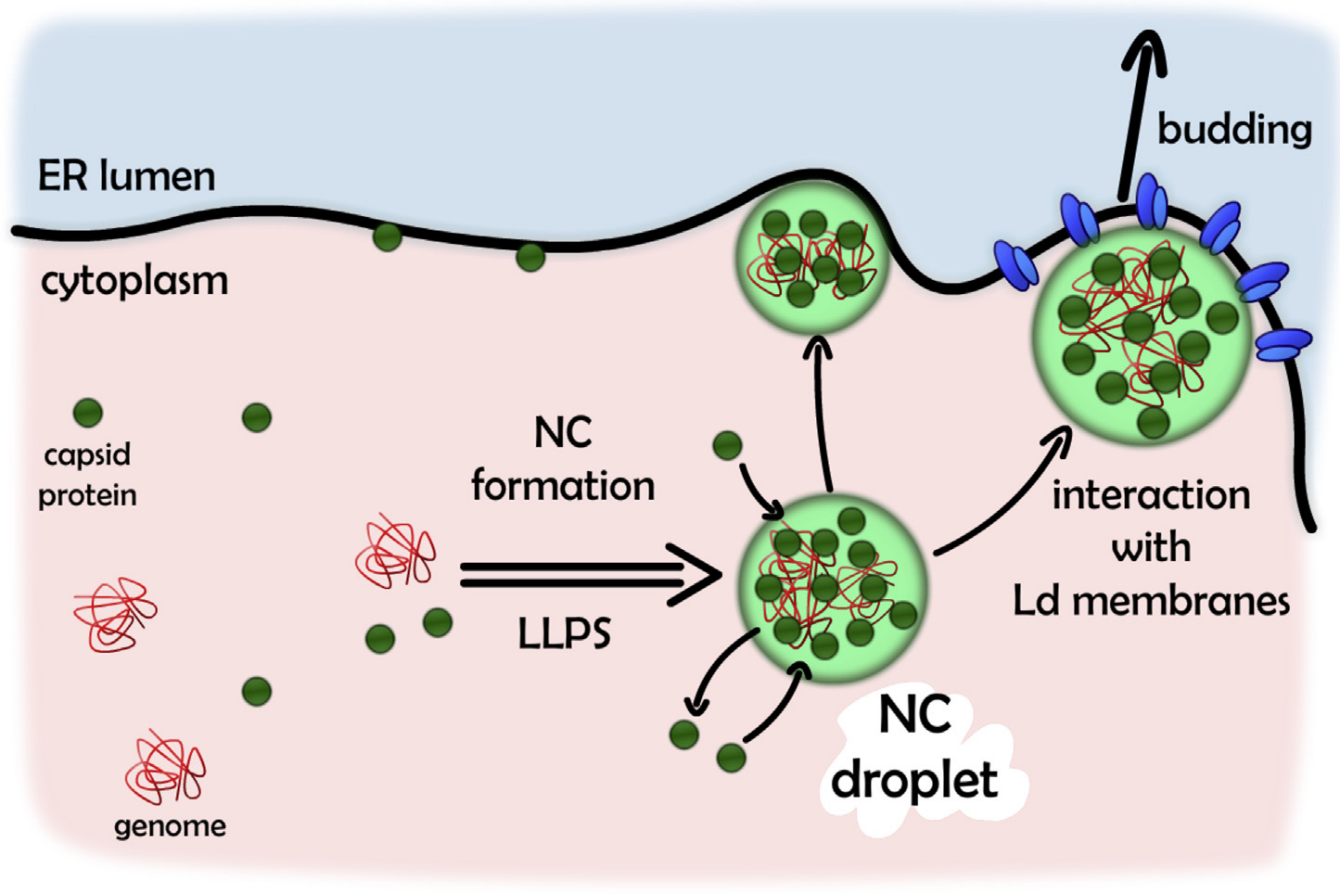
**Schematic representation of the encapsidation indicating a process of liquid–liquid phase separation (LLPS).** The interaction between capsid protein (*green circles*) and the viral genome (*red*) results in a liquid–liquid phase transition to form the NC structure (*light green droplet*). These droplets associate with the ER membranes during the viral particle formation stabilizing an NC–membrane interaction for final viral particle budding into the ER lumen. ER, endoplasmic reticulum; NC, nucleocapsid.

Altogether, we provide compelling data on the physicochemical properties of complexes that include membranes, viral proteins, and nucleic acids, proposing a new role for liquid droplet formation and membrane docking on the viral NC assembly, as schematized in Figure 5. Considering the virus encapsidation process not as a result of mere molecular interactions but as an emerging new liquid phase might help to clarify other important aspects of the virus development. For example, the NC structure also has a role in protecting the integrity of the viral genome, which is very important for the virus survival. To phase separate the viral RNA inside the cell can contribute to exclude cellular components that would compromise its stability and avoid damage. Actually, the fact that the water activity is different in this new phase, as we show here, could have as a consequence a possible reduction of water-dependent enzymatic degradation, for instance, by hydrolytic RNases (25, 26). Therefore, understanding the capsid capacity to bind membranes and recruit genomic information into the lipid interface in a new liquid phase, a novel scenario of possible targets to impair viral particle maturation is open. In this sense, the development of new antiviral therapies can be focused on the disruption of the capsid–ER or capsid–RNA association and liquid condensation.

## Experimental procedures

### Reagents

Oligonucleotides ssDNASel25 (5′GGACAGGAAUUAAUAGUAGCUGUCC3′) and FITC(5′)-ssDNASel25 oligonucleotides were obtained from Invitrogen, Cy5-DNA (5′ Cy5-AAACTATTATTACTCATTTTCCGCCAGCAGTCAACTTCGATTTAATTCGTAAACAGATCT3′) was purchased from Macrogen, and Cy5-RNA (5′ Cy5-CAUCAUGCAGGACAGUCGGAUCGCAGUCAG 3′), Cy3 RNA (5′ CUGUCCUGCAUGAUG-Cy3 3′), and functional RNA (5′ FAM-AGUUGAGUUGAGUUG 3′) were purchased from IDT. For the experiments presented in this work, the oligonucleotides were diluted in DNase-free and RNase-free water.

All lipids used in the experiments (10 mg/ml) including egg NBD–PE (l-α-PE-*N*-(7-nitro-2-1,3-benzoxadiazol-4-yl); 1 mg/ml) were purchased from Avanti Polar Lipids, Inc. TRITC–PE (TRITC-1,2-dihexadecanoyl-*sn*-glycero-3-phosphoethanolamine, 1 mg/ml in 2:1 chloroform:methanol) is from Invitrogen and naphthopyrene from Sigma–Aldrich. ACDAN was purchased from Santa Cruz Biotechnology, Inc. All stock solutions were prepared in chloroform:methanol 2:1 (v:v) at concentrations ranging 20 to 1 mg/ml and kept at −80 °C. All laboratory reagents (including buffers and salts) are from Sigma and Merk and used without further purification. Water was either milli-rho or Milli-Q quality, obtained from an ion-exchange resin.

### Proteins

Constructs encoding capsid proteins were derived from the complementary DNA of an infectious clone of DENV2 strain 16681 (GenBank accession number: U87411) and the complementary DNA of a lab-adapted virus from Senegal, A1C1/V2 (GenBank accession number: KX198134) from ZIKV. The coding sequence of DENV capsid protein was amplified from residues 1 to 100 by PCR and cloned into a pET15b vector between NcoI and BamHI to generate plasmid pET-DENVC. The coding sequence of ZIKV capsid protein was amplified from residues 1 to 104 by PCR and cloned into a pET15b vector between NcoI and BamHI to generate plasmid pET-ZIKVC.

For protein expression, competent Rosetta BL21 (DE3 pLys) *Escherichia coli* bacteria were transformed with the aforementioned expression plasmids. Transformed cells were grown at 37 °C in LB medium with ampicillin (100 μg/ml) and chloramphenicol (25 μg/ml) to an absorbance of 0.6 (600 nm wavelength) and then induced with IPTG at a final concentration of 0.5 mM. The induced cells were incubated at 20 °C for 5 h, and the cells were then harvested by centrifugation at 4000 rpm for 10 min. The cell pellet was resuspended in lysis buffer containing 50 mM NaH_2_PO_4_/Na_2_HPO_4_, 0.1 M NaCl, 20% glycerol, 1% Triton X-100, Protease Inhibitor Cocktail (Sigma), and 5 mM 2-mercaptoethanol. The suspension was sonicated, clarified by centrifugation, and then filtered with a 0.45-μm syringe filter. The cell extract was applied to a HiTrap Heparin HP column (GE Healthcare) equilibrated in 50 mM NaH_2_PO_4_/Na_2_HPO_4_ buffer with 2% glycerol and washed and eluted with the same equilibration buffer with increasing concentrations of NaCl. The fractions containing the capsid protein were pooled and concentrated using an Amicon Ultra-15 centrifugal filter (Merck Millipore), and the buffer was exchanged to a final storage buffer composed of 50 mM NaH_2_PO_4_/Na_2_HPO_4_. The protein was stored at −20 °C.

A cysDENV (N93C) cysteine mutant of DENV capsid was generated by replacing the asparagine at the position 93 by cysteine. The mutation was incorporated in the reverse primer used to amplify the coding sequence of DENV capsid protein by PCR. The mutated protein was cloned, expressed, and purified as previously described. This mutant allows us to label the capsid protein with a maleimide-derivative dye. In our case, we used Alexa^488^-maleimide to label cysDENV and to be able to detect the protein directly thorough the fluorescence microscope. Protein labeling was carried out by adding 20 μl of 0.1 mg/ml Alexa^488^-maleimide in dimethyl sulfoxide into a cysDENV aliquot, to a final dye/protein ratio of 5. The suspension was incubated up to 1 h, and the noncoupled free dye was removed by exclusion chromatography using a PD10 exclusion column (GE Healthcare) equilibrated with 50 mM NaH_2_PO_4_/Na_2_HPO_4_ with 1% glycerol.

### Liposomes

Liposomes were mainly composed of a lipid mixture (ERmix), resembling the ER lipid composition, following that reported by Keenan and Morré (27). Specifically, the following was the composition of the ERmix lipid used (mole %): 4% brain sphingomyelin, 60% egg phosphatidylcholine, 3% liver phosphatidylserine, 9% liver phosphatidylinositol, 9% liver PE, and 15% cholesterol. L_o_/L_d_ GUVs were generated as described by Baumgart *et al.* (28) with a 30% 1,2-dioleoyl-*sn*-glycero-3-phosphocholine, 15% 1,2-dioleoyl-*sn*-glycero-3-phosphoserine, 15% cholesterol, and 40% brain sphingomyelin lipid mix.

GUVs were obtained following the procedure described by Weinberger *et al.* (29). Briefly, 200 μl of a 5% (p/v) of polyvinyl alcohol (water solution) were spread onto a clean coverslip and dried for 1 h at 70 °C. Coverslips were cleaned using a 1% Hellmanex II (Sigma–Aldrich) water solution for 3 h. Once dried, 20 μl of the specific lipid mix in chloroform:methanol 2:1 at 0.5 mg/ml and doped with 0.5 to 2% fluorescent lipophilic dyes (*i.e.*, TRITC–PE and naphthopyrene) were dispersed onto the polyvinyl alcohol film with a Hamilton syringe in a dropwise manner. To eliminate the organic solvent, the sample was first evaporated under a nitrogen stream followed by a treatment in a high vacuum chamber for 2 h. GUVs were prepared by addition of 200 μl of a sucrose solution on the surface of the dried polymer cushion, which contains the lipids. Osmolarities of the sucrose solution range from 290 to 310 mOsM as checked in a freezing point osmometer from Löser Messtechnik. After 45 min of the sucrose addition, GUVs are harvested by aspiration with a micropipette, kept in a plastic microtube, and used the same day.

For the preparation of LUVs (30), the appropriate amount of lipids was mixed in conic glass tubes, slowly dried with a steam of nitrogen gas, and left in vacuum for 2 to 3 h. Dried lipids were supplemented with HKM buffer (20 mM Hepes buffer with 120 mM potassium acetate and 1 mM MgCl_2_) to a final total lipid concentration of 4 mM. This lipid suspension was frozen and thawed five times using nitrogen (l), and a thermal water bath set to 37 °C, respectively. After the last freeze–thaw cycle, the sample was extruded 20 times through a 100-nm pore size polycarbonate membrane from Whatman (Schleicher & Schuell) at 23 to 25 °C in Avestin extrusion device.

### Techniques

#### Fluorescence measurements

##### Interactions of DENV and ZIKV capsids with LUVs

All fluorescence measurements of this section were conducted on a Cary Eclipse Spectrofluorometer using a 3-mm optical path length cell and at 23 to 25 °C. The W fluorescence emission spectra of the proteins, in the absence and in the presence of ERmix LUVs (1 mM), were obtained from a 5 μM protein solution in HKM buffer. W excitation was at 280 nm wavelength, and the emission spectra were observed at the 310 to 420 nm range.

To study the binding of the capsid proteins to LUV, FRET experiments between the protein W and the lipophilic probe NBD–PE were performed accordingly (12). Briefly, 200 μM ERmix LUVs suspension, doped with 2% NBD–PE, in HKM buffer was titrated with different amounts of the proteins, and the NBD fluorescence emission (acceptor) was collected at 530 nm exciting W at 280 nm (donor fluorophore). The maximum protein to total lipid mole ratio reached is 0.05.

To evaluate a potential exchange of lipids among different liposomes, promoted by the interaction of the proteins with the membranes, we use another FRET couple. Briefly, 0.2 mM ERmix LUVs presenting 2% mole NBD–PE (donor fluorophore) and 3% mole TRITC–PE (acceptor fluorophore) were mixed with 0.8 mM of nonstained LUVs in HKM buffer. NBD fluorescence emission was registered at 530 nm with an excitation wavelength of 467 nm. If lipids from labeled and nonlabeled liposomes are mixed upon protein addition, the donor–acceptor collisional probability decreases resulting in a net donor fluorescence increase (31). Experiments were performed by recording the donor signal in the absence and presence of either 5 μM DENV or ZIKV capsid proteins (0.005 protein to total lipid mole ratio). Control experiments representing a total separation of the NBD–TRITC–PE FRET couple were performed by addition of Triton X-100 (0.5% v/v final concentration).

##### Nucleic acid–protein interaction characterization by fluorescence anisotropy

Steady-state anisotropy measurements of Cy5-labeled DNA or Cy5-RNA were carried out on an ISS PC1 spectrofluorometer (ISS, Inc). Excitation of Cy5 was performed with a 635-nm laser diode, and emission was collected through a 720-long pass filter. The excitation and emission slits used were 1 mm, which give a bandpass of 8 nm. At the maximal protein concentration used (up to 2.5 μM), no significant background because of scattered light or buffer (HKM buffer) fluorescence is detected. Every anisotropy point, from free oligonucleotide (80 nM) until the last protein addition, results from the average of ten continuous measurements at 22 °C.

The gaseous equilibrium adsorption process between two phases and the molecular aggregation effect on the ligand binding were described by the seminal works of Langmuir and Hill, respectively (32, 33). We used the Hill adsorption isotherm to analyze the thermodynamics and the cooperativity of oligonucleotide–protein interaction (33). The fraction, *θ*, of ligand-binding sites on the protein that are occupied by a ligand (L) is described as:

(1) $$\theta =\frac{{\left[L\right]}^{n}}{{\left[L\right]}^{n}\;+\;K_{d}}$$

Where *n* is represented to be the binding site number but, experimentally, is a measure of the degree of interaction between these sites and [L] the concentration of free ligand (34). *θ* can be related to the concentration of [*LR*] as $\theta =\frac{\left[LR\right]}{R_{T}}K_{d}$, being *R*_*T*_ the total protein concentration. With this expression, a plot of [*LR*] *versus* protein concentration is used to fit the model:

(2) $$\left[LR\right]=\frac{R_{T}{\left[L\right]}^{n}}{K_{d}^{2}\;+\;K_{d}{\left[L\right]}^{n}}$$

From our anisotropy data, we estimated [*LR*] as the total oligonucleotide *L*_*T*_ minus the free ligand fraction determined as $\frac{v_{obs}-v_{max}}{v_{min}-v_{max}}L_{T}$, where *v*_*obs*_, *v*_*min*_, and *v*_*max*_ are the observed, minimum, and maximum anisotropy values, respectively. Equation 2 was fitted to the experimental data by using OriginPro 8 (OriginLab Corporation).

##### Fluorescence confocal microscopy and image analysis

Samples were studied with spectral Olympus FV1000 or FV1200 laser scanning confocal microscopes equipped with different lasers lines (FV1000: 405, 488, 561, and 633 nm; FV1200: 405, 473, 559, and 635 nm) and objectives (FV1000: 63×, 1.4 numerical aperture; FV1200: 60×, 1.42 numerical aperture) in a room with a temperature set at 23 °C. In general, sequential images at a fixed *z* position were acquired. Samples were observed either in 8-well Lab Tek chamber slides (Promega) or a home-made Ludin chamber. Before observation, coverslips were passivated with casein 1% in HKM buffer for 20 min and rinsed with HKM.

Experiments with GUVs were normally carried out by adding 1 to 2 μl of the stock of GUVs into a 0.6-ml plastic microtube and then incubated with 0.5 to 3 μM of proteins in HKM buffer. Depending on the experiment, a sequential addition of the other reagents (oligonucleotides and/or LUVs) was done. The working final volume was always 200 μl. The sample was added into the microscope observation chamber, and GUVs were imaged after 15 min to ensure liposome sedimentation because of their sucrose content.

The excitation wavelengths for the different fluorophores used in our microscopy experiments were 405 (ACDAN and pacific blue), 488 nm (Alexa^488^, FITC, and naphthopyrene), 559 nm (TRITC), and 635 nm (Cy5).

The time sequence of the incorporation of Alexa^488^-DENV into pre-existing liquid droplets was conducted by incubating 3 μM DENV + 3 μM Sel25 DNA for 15 min (droplet formation) directly in the observation Ludin chamber, starting the time-lapse fluorescence confocal acquisition of four *z* planes with a 0.5 μm z spacing and then injecting 8 μl of Alexa^488^-DENV 12 μM (reaching 0.5 μM final concentration). Every time point (4 s) is the built up from the maximum intensity image projections. Once the time stack image was obtained, we used the particle analysis tool of the ImageJ software (National Institutes of Health), thresholding the stack to individualize the 90% of the particles and quantify the Alexa^488^ time-dependent signal increase at every single droplet.

Information of water dynamics at the oligonucleotide–protein stabilized droplets was obtained using the fluorescence probe ACDAN. As previously described (18), this probe responds to the extent of water relaxation occurring in its milieu. Specifically, as a first step, we incubated either 3 μM DENV or 3 μM ZIKV with 3 μM Sel25 DNA for 15 min directly in the observation chamber (200 μl). Then, the fluorescent ACDAN dye was added from an ethanolic stock solution (1.4 mM) to a final concentration of 14 μM. The confocal spectral imaging experiments were performed by exciting at 405 nm and collecting the emission light between 450 and 732 nm with a resolution of 3 nm. For these experiments, we used clean noncasein passivated coverslips in order to reduce possible interactions between ACDAN and the coverslip surface. Fluorescence emission spectra obtained from the spectral images at selected regions of interest were normalized to allow better comparison.

## Data availability

All data supporting this article will be shared upon request to Dr Ernesto E. Ambroggio, eambroggio@unc.edu.ar.

## Supporting information

This article contains supporting information.

## Conflict of interest

The authors declare that they have no conflicts of interest with the contents of this article.
