# Supplementary material for: Dengue and Zika virus capsid proteins bind to membranes and self-assemble into liquid droplets with nucleic acids
Source: J Biol Chem. 2021 Aug 8;297(3):101059. doi: 10.1016/j.jbc.2021.101059 (PMC8397897; doi:10.1016/j.jbc.2021.101059)
Supplement: Supporting information [file mmc1.pdf]

Supporting Information for

Dengue and Zika virus capsid proteins bind to membranes and self-assemble into liquid droplets with nucleic acids

Ernesto E. Ambroggio*, Guadalupe Soledad Costa Navarro, Luis Benito Pérez Socas, Luis A. Bagatolli, Andrea V. Gamarnik*.

*To whom correspondence should be addressed:
Email: eambroggio@unc.edu.ar; agamarnik@leloir.org.ar

DENV and ZIKV capsid interaction with liposomes: Liposome Electrophoretic Mobility Shift Assay (LEMSA) of and MLVs sedimentation

The figure S1 and table S1 show a LEMSA assay for the binding of DENV and ZIKV capsid proteins to ER_{mix} LUVs. This result shows the electrophoretic mobility of the liposomes changes in presence of the proteins. The assay was performed according to (1). Briefly, NBD-PE labelled ER_{mix} liposomes were incubated with the indicated concentration of unlabeled proteins and loaded in the middle point of a 0.35% agarose gel. An electrophoresis experiment is carried out at 100 V of constant voltage and images are taken every ten minutes. The observed bands correspond to the liposomes and measurements of the band displacement over time allows to calculate the electrophoretic mobility value (μ, see the table A). The example in the figure is taken from a 30-minute run. Experiment was done in duplicate, and the table shows the mean and standard deviation for μ.
These liposomes bear a negative charge density and a migration toward the positive pole is observed when there is no protein in the sample. However, when the protein is present is possible to observe a population of liposomes migrating toward the negative pole, indicating a change in polarity once the protein is bound to the vesicles.
These data are now submitted as Supporting Information together with the new version of the manuscript.

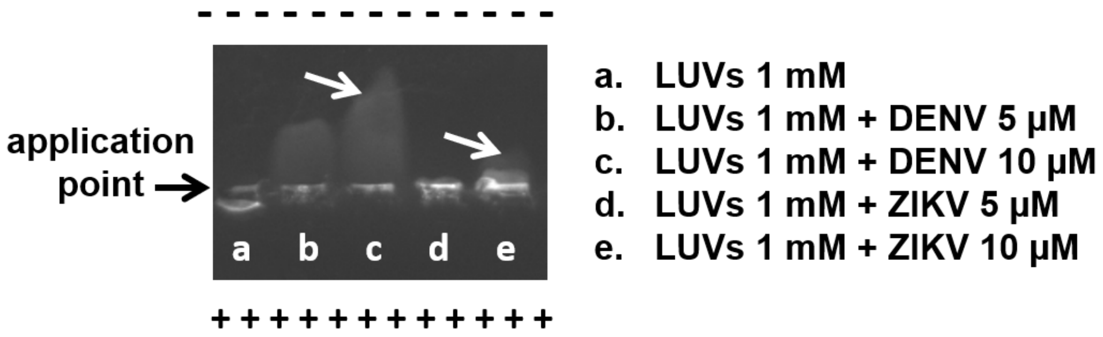

	μ (10^{-5} $cm^2V^{-1}s^{-1}$)
LUVs 1 mM	-1.0 ± 0.2
LUVs 1 mM + DENV 5 μM	2.23 ± 0.04
LUVs 1 mM + DENV 10 μM	2.40 ± 0.03
LUVs 1 mM + ZIKV 5 μM	1.0 ± 0.3
LUVs 1 mM + ZIKV 10 μM	1.22 ± 0.07

Figure S1 and Table S1. LEMSA assay of NBD doped ERmix LUVs in the absence (line a) or in the presence of DENV (line b and c) and ZIKV (line d and e) capsid proteins. In table 1 we report the electrophoretic mobility value, μ, for each condition.

Figure S2depicts a lipid pull-down experiment indicating a specific binding of a capsid protein to ERmix multi-lamellar vesicles (MLVs). Briefly, MLVs (200μM total lipid) where generated by lipid hydration in HKM buffer and incubated for 15 min at room temperature with 5 μM of the capsid protein. Afterwards, the mixture was centrifuged, and the pellet (MLVs) and supernatant fractions were collected. As can be observed, when the MLVs were made of the ER lipid mix, the protein band is clearly found in the pellet fraction, P, confirming a physical interaction of the protein with the lipid composition used in our work. On the other hand, we evaluated MLVs composed of POPC (a zwitterionic non-ER mimetic lipid). The result is sharp since the proteins are now only detected in the supernatant (SN) fraction indication no binding to the POPC MLVs.

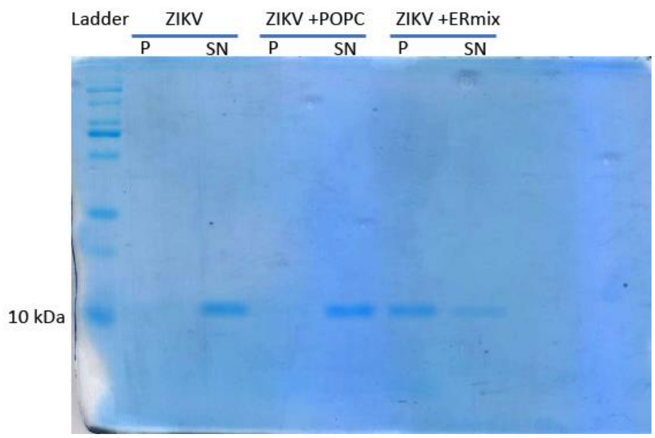

Figure S2. SDS-PAGE assay of a multilamellar vesicles (MLVs) pull down experiment in the presence of ZIKV capsid protein. The gel vertical lines show the molecular weight ladder (Ladder) and the pellet (P) and supernatant (SN) fractions after centrifugation of pure protein (ZIKV), protein + POPC MLVs (ZIKV +POPC) and protein + the ERmix MLVs (ZIKV +ERmix).

Alexa^{488}-DENV binds ERmix GUVs and recruits RNA and LUVs to the membrane.

Figure S3 shows additional data on how Alexa488-DENV is able to interact with ERmix GUVs and can recruit fluorescently labelled RNA and LUVs to the interface of the giant liposomes. As can be observed, a differential spreading of all the components onto the GUVs membrane is only observed when Alexa^{488}-DENV is present.

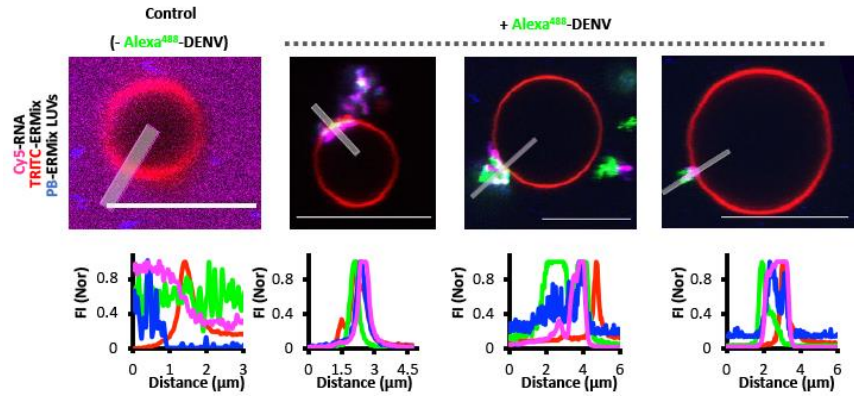

Figure S3. Additional color-merged confocal fluorescence images of TRITC-PE ERmix GUVs (red channel) incubated with Cy5-RNA (magenta channel) and with PB labelled ERmix LUVs (blue channel) in the absence (control condition; - Alexa^{488}-DENV) or in the presence of Alexa^{488}-DENV (green channel; + Alexa^{488}-DENV). Scalebar in confocal images scales to 10µm.

References

1. Socas, L. B. P., and Ambroggio, E. E. (2020) THE INFLUENCE OF MYRISTOYLATION, LIPOSOME SURFACE CHARGE AND NUCLEIC ACID INTERACTION IN THE PARTITION PROPERTIES OF HIV-1 GAG-N-TERMINAL PEPTIDES TO MEMBRANES. *Biochim. Biophys. Acta - Biomembr.*
